# A Case of Rupture of the Uterus

**Published:** 1862-01

**Authors:** E. L. Holmes

**Affiliations:** Chicago


### CHICAGO MEDICAL JOURNAL.

VOL. V.] JANUARY, 1862. [No. 1.

### ORIGINAL COMMUNICATIONS.

### A CASE OF RUPTURE OF THE UTERUS.

By E. L. HOLMES, M. D., of Chicago.

The statistics of obstetric science, derived from hospital records, present so large an aggregate number of cases of Rupture of the Uterus, and our periodical publications give us so frequently the history of such cases, as they occur in private practice, that the report of an additional case may possibly be considered by our readers as useless.

The following brief report, however, of a case of this terrible accident may be of some importance to those interested in collecting facts relating to this subject:

I was called some time since to attend an Irish woman about 32 years of age, in her fourth confinement. The liquor amnii had been discharged about fourteen hours before my visit, although decided labor pains did not commence till ten or twelve hours after the discharge of the uterus. The patient informed me that her health had always been good even in pregnancy, but that her previous labors had been protracted and quite difficult.

The os uteri was but slightly dilated—head presenting with vertex to the left acetabulum—pains very moderate and occurring at intervals of ten or fifteen minutes.

WI
CH561

At the end of six hours, during which time I had visited the patient quite often, the os uteri had become well dilated and the head had passed the superior strait. The patient was in good spirits and quite strong. Labor was tedious, but otherwise progressing favorably.

I had occasion to leave the house for a few moments, and on my return was informed at the door that the patient was dying. It appeared that a moment before, while in an ordinary pain, the patient suddenly uttered a shriek and fainted, when all symptoms of labor pains ceased. I found her complaining of intense pain in the abdomen and of great thirst. The extremities were cold, lips purple and face covered with cold prespiration. The breathing was hurried, pulse scarcely perceptible and strength evidently failing. I immediately removed the pillow from the head; directed full doses of brandy and water, and sent a messenger a short distance for Prof. Freer, who arrived in a few minutes; and, as a pair of forceps was not at hand, delivered the woman with some difficulty by turning. On passing my hand into the uterus for the placenta, I found the organ wholly uncontracted, with a rupture in the fundus about 4½ inches long, extending obliquely across the right side, downwards and forwards. A fold of the intestines was lying in the rupture. There was no more external hæmorrhage than usual, nor was there an undue amount of coagula in the uterus with the placenta.

The great prostration of the patient was undoubtedly caused by the nervous shock rather than the extensive loss of blood. Death occurred about three minutes after the delivery of the child. No autopsy was obtained.

